# Assessment of ‘Golden Delicious’ Apples Using an Electronic Nose and Machine Learning to Determine Ripening Stages

**DOI:** 10.3390/foods13162530

**Published:** 2024-08-14

**Authors:** Mira Trebar, Anamarie Žalik, Rajko Vidrih

**Affiliations:** 1 Faculty of Computer and Information Science, University of Ljubljana, Večna pot 113, 1000 Ljubljana, Slovenia; 2 Biotechnical Faculty, University of Ljubljana, Jamnikarjeva 101, 1000 Ljubljana, Slovenia; anamariezalik@gmail.com (A.Ž.); rajko.vidrih@bf.uni-lj.si (R.V.)

**Keywords:** electronic nose, ‘Golden Delicious’ apple, ripening stages, PCA, K-means clustering, KNN

## Abstract

Consumers often face a lack of information regarding the quality of apples available in supermarkets. General appearance factors, such as color, mechanical damage, or microbial attack, influence consumer decisions on whether to purchase or reject the apples. Recently, devices known as electronic noses provide an easy-to-use and non-destructive assessment of ripening stages based on Volatile Organic Compounds (VOCs) emitted by the fruit. In this study, the ‘Golden Delicious’ apples, stored and monitored at the ambient temperature, were analyzed in the years 2022 and 2023 to collect data from four Metal Oxide Semiconductor (MOS) sensors (MQ3, MQ135, MQ136, and MQ138). Three ripening stages (less ripe, ripe, and overripe) were identified using Principal Component Analysis (PCA) and the K-means clustering approach from various datasets based on sensor measurements in four experiments. After applying the K-Nearest Neighbors (KNN) model, the results showed successful classification of apples for specific datasets, achieving an accuracy higher than 75%. For the dataset with measurements from all experiments, an impressive accuracy of 100% was achieved on specific test sets and on the evaluation set from new, completely independent experiments. Additionally, correlation and PCA analysis showed that choosing two or three sensors can provide equally successful results. Overall, the e-nose results highlight the importance of analyzing data from several experiments performed over a longer period after the harvest of apples. There are similarities and differences in investigated VOC parameters (ethylene, esters, alcohols, and aldehydes) for less or more mature apples analyzed during autumn or spring, which can improve the determination of the ripening stage with higher predicting success for apples investigated in the spring.

## 1. Introduction

Apples are stored at low temperatures (0–4 °C) under controlled atmosphere conditions before being made available to consumers in retail stores. In retail stores, apples are kept at ambient temperatures for the ‘unknown period’, and after purchase, they spend additional time at different temperatures in the consumer’s home. The duration from arrival to store until consumption is so-called ‘shelf life’. Extensive research and analyses of fruit quality during shelf life have been published. During the shelf life, the quality of apples may change substantially, so quality control is necessary.

Consumers might pay more for premium quality, so it is very important to have sufficient information regarding the history of apples, such as traceability (agricultural practices, storage, and fruit handling) [1]. At the point of sale, factors such as freshness, taste, safety, and sensory quality are directly related to ripening stages [2]. This classification is mainly provided by inspection carried out by experts, which can be an error-prone process. Recently, numerous automated approaches have been introduced, including complex laboratory and electronic systems for analyses and data collection, and machine learning and deep learning methods to monitor and inspect agricultural products in relation to aroma and VOCs [3].

The main aim of this study focuses on the analysis of ‘Golden Delicious’ apples based on experiments performed with an electronic nose over two years. The analysis includes the spring and autumn periods of apples received directly from the producer and purchased in supermarkets. Concentrations of Volatile Organic Compounds (VOCs), measured with four MOS sensors, were used in statistical analysis and a machine learning approach to classify any new investigated apples into one of three ripening stages (less ripe, ripe, and overripe). Furthermore, this study also investigated the selection and importance of sensors used in the electronic nose. The novelty of the presented study could be emphasized with the following findings:

- Use of an electronic nose provides a non-destructive and user-friendly approach to achieve better accuracy of quality control in supermarkets.
- Detection of seasonal variations in VOCs can improve the determination of ripening stages between less and more mature apples.
- Using data from multiple experiments performed over a longer period provides robustness of findings to be more applicable to real-world scenarios.
- A multimethod approach improves classification accuracy using PCA and K-means clustering to identify three ripening stages used for the evaluation of the KNN model.

## 2. Related Studies

Quality and maturity stages of ‘Golden Delicious’ apples have been addressed in numerous studies [4,5,6,7]. These stages can be easily determined and classified based on color and visible defects, employing colorimetric parameters such as CIE L*, a*, and b*, or by assessing respiration and ethylene production rates. Volatile organic compounds (VOCs) are also crucial quality parameters; their emission depends on the harvest time, storage conditions, and possible treatment with 1-Methylcyclopropene (1-MCP) [7]. Some studies have identified key aroma compounds [8], such as esters and alcohols, in apple juice. Controlled atmosphere storage conditions inhibited the production of esters like butyl and hexyl acetate.

During the storage period of ‘Golden Delicious’ apples, temperature significantly affects weight loss [9,10]. The results indicate that significant alterations were observed at ambient temperature (approximately 25 °C) compared to the recommended storage temperature (5 °C). Apples cultivated in Portugal were analyzed weekly for three months in normal atmospheric conditions (0.5 °C, 90% RH) immediately after the harvesting period, revealing that firmness, soluble solids, and starch index were notably influenced by storage time. In a study on the shelf life of apples after a late harvest, stored at ambient temperature for six weeks [11], an increase in total soluble solids (TSS) and weight loss accompanied by color darkening was observed. Firmness and VOCs were investigated in a three-year study carried out in two regions of the United States on Golden and Red Delicious apples harvested weekly from August to October and were found to be dependent on the pedoclimatic conditions of their cultivation [12].

Various destructive and non-destructive methods are available for determining the ripening stages of fruit. Many of these methods are complex and require analysis in the laboratory, often involving expensive equipment. Additionally, sensory evaluation demands a stable, trained panel to validate pear genotypes for determining the relationship between sensory data and instrumental data (firmness, total soluble solids (TSS), and titratable acidity (TA)). This process often involves destructive methods, such as the preparation of samples that can only be performed once on the same sample. Moreover, sensory experts may need additional training, as exemplified in the case of sensory attributes for pears [13].

A machine vision system was employed for grading the ‘Golden Delicious’ apples in six steps [14]. The color image of the apple undergoes preprocessing and is then analyzed using machine learning approaches (Support Vector Machine—SVM, Multilayer Perceptron—MLP, and K-Nearest Neighbors—KNN) to classify the apple into two categories (healthy and defected) and three quality categories (first rank, second rank, and rejected). The Support Vector Machine (SVM) classifier yielded the best results, with a recognition rate of 92.5% for the two categories and 89.2% for the three categories. The delta absorbance (DA) meter (non-destructive) was used to measure the chlorophyll content of ‘Golden Delicious’ apples at harvest and during the ripening process [15]. Additional tests of firmness and the inhibition of ethylene production were performed to assess the correlation between results of quality attributes after commercial storage based on I_AD_ (index of absorbance difference). In Mexico, apple production faces significant losses during processing, making it essential to monitor external color and physicochemical properties for ripening stage classification [16]. The ripening index (RPI) with three stages (unripe, ripe, and senescent) was compared to color variability using colorimetric parameters (CIE L*, a*, and b*), color space (Chroma C), and hue angle (h*) with PCA analysis used to evaluate the correlation between variables. Fruit ripening classification [17] was performed using a mobile application for a group of four fruits (red and green apple, banana, strawberry, and orange) in three maturity stages (immature, mature, and rotten). This classification was based on training a convolutional neural network using a dataset of images.

To maintain the quality of stored apples [18], the ethylene hormone was analyzed using laser photoacoustic spectroscopy, and ripening and external defects were detected through image and spectrum analysis. The study found a negative correlation between the ethylene evolution and ethanol content, which is associated with the senescence of apples. Identification of quality, fruit defects, and diseases is crucial for increasing efficiency and reducing losses during and after harvest [19]. In the review, the non-destructive method utilizing X-ray imaging technology in conjunction with an artificial network was employed in the analysis of mango. Additionally, Principal Component Analysis (PCA), Linear Discriminate Analysis (LDA), and Competitive Learning Neural Network using Fuzzy image processing were used in the sorting process of mangoes. An analysis of physical and chemical attributes (firmness and total soluble solids (TSS)) along with sensory evaluation of apples kept at ambient temperature showed the acceptability of the grading and sorting process [20]. The quality assessment of agricultural products is becoming increasingly important in waste management [21]. Image classification employing Convolutional Neural Network (CNN) was used to achieve the ripening grading of hawthorn fruit. The prediction of fruit spoilage was analyzed using laser spectroscopy in sealed packaging during storage [22]. Quantifying the volatile compounds of grapes helped detect the spoilage early enough to predict the storage time.

Significant developments in electronic noses since the 1980s are attributed to the new technologies and improvements in sensor design. Research on various systems to analyze the maturation of apples has become available. The portable electronic nose (PEN 2) was evaluated to discern differences between ripening stages (commercial ripe, ripe, and overripe) of cherries from various producers [23]. Additionally, PEN 3 was used to construct a shelf life prediction model for ‘Golden Delicious’ apples [24]. Furthermore, various chemical and MOS sensors have facilitated sustainable, non-destructive monitoring, and process control [25]. The availability of various types of data, new applications, and approaches enabled by electronic noses, along with pattern recognition and classification methods, has advanced product quality attributes with a range of gas and chemical sensors [26,27]. Principles, usefulness, and limitations were addressed and compared in connection with pattern recognition algorithms (ANN, CNN, PCA, PLS, and SVM), emphasizing the importance of strict sample preparation, data sampling and processing, and addressing poor repeatability. Several research publications have analyzed different fruits using electronic noses [28,29,30], employing various MOS sensors (MQ3, MQ4, and others). Changes in the production of VOCs were evaluated through computational approaches using PCA, LDA, SIMCA, and SVM. The studies show an SVM classification model of banana ripening stages with an accuracy of 98.66% and several prediction models successfully distinguishing between fresh or spoiled stone fruit [26]. Simple, rapid, and inexpensive detection methods with sensors significantly contribute to the validity and correctness of collected data.

In the assessment of fruit aroma, electronic noses equipped with a large number of sensor arrays were investigated to discern the chemical nature of volatile compounds in various fruit types [31,32]. For climacteric fruit, ethylene is the key component in fruit maturation used to detect the ripeness stage. In the continuous monitoring of mango fruit, the method successfully differentiated between three ripeness stages (unripe, ripe, and overripe). Employing Metal Oxide Semiconductor (MOS) sensors to detect VOCs, the Artificial Neural Network (ANN) effectively classified fruit samples of apples, bananas, oranges, grapes, and pomegranates into unripe, ripe, or overripe stages with an average accuracy equal to or higher than 95% [33]. The quality of nectarines and peaches [34] was presented with Principal Component Analysis (PCA) and Multiple Linear Regression (MLR) to build a predictive model for the attribute aroma, along with acidity, sweetness, and acceptability. Maintaining the quality related to ripening during the distribution of durian fruit was evaluated with gas sensors and neural networks based on the aroma [35], achieving a 91% probability.

## 3. Materials and Methods

### 3.1. Electronic Nose

**Figure 1:**
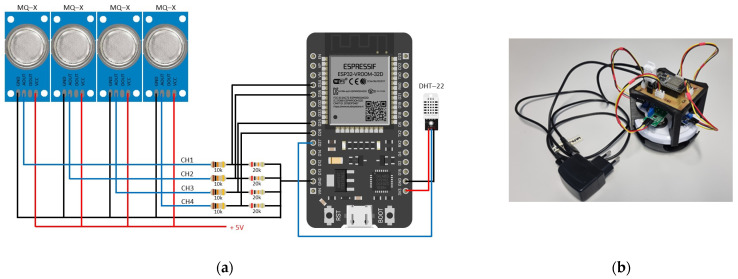
Electronic nose: (**a**) connection of sensors to ESP32-DevKitC V4 Board and (**b**) prototype of electronic nose placed on the jar lid.

‘Golden Delicious’ apples were provided directly from a storage house (conditions: +1 °C, 1% O_2_, 1.2% CO_2_, and >92.0% relative humidity) or from a retail shop (room temperature, approximately 50% relative humidity). In the experiments performed for the ripening analysis of apples stored in ambient conditions, a prototype of an electronic nose was employed [36]. It comprises four MOS (Metal Oxide Semiconductor) sensors, which are controlled by an ESP32-DevKitC V4 Board [37] (Figure 1a). The electronic nose is connected to the computer via Mini–B USB (Figure 1b), where an application runs to collect sensor measurements according to the programmed controller with a specified time interval.

Additionally, a temperature and relative humidity module DHT22 [38] is used to measure environment conditions during the experiment, with temperature ranging from −40 to +80 °C (±0.5 °C) and relative humidity ranging from 0 to 100% (5% accuracy).

Four MOS sensors produced by Winsen Electronics Technology Co., Ltd., Zhengzhou, China, used in the electronic nose, with their target odors and sensitivity ranges are detailed in Table 1. The MQ3 gas sensor [39] is designed to detect alcohol vapors, including methanol, ethanol, and isopropanol, making it useful for alerting to the presence of alcohol in the environment. MQ135 gas sensor [40] is well-suited to detect ammonia (NH_3_), nitric oxide (NOx), alcohol, benzene, smoke, and CO_2_, making it suitable for air quality control applications. MQ136 gas sensor [41] is highly sensitive to hydrogen sulfide (H_2_S) and can detect vapors containing sulfur, rendering it suitable for domestic and industrial applications. MQ138 gas sensor [42] detects toluene, acetone, ethanol, and hydrogen. It can also detect organic volatile compounds, and its low-cost design makes it suitable for various applications in VOC gas alarms and gas leakage detection. These four sensors were chosen based on the results in [29], as they were identified as the most effective to detect VOCs, usually determined as fruit aromas and alcohols.

### 3.2. Experimental Setup

**Figure 2:**
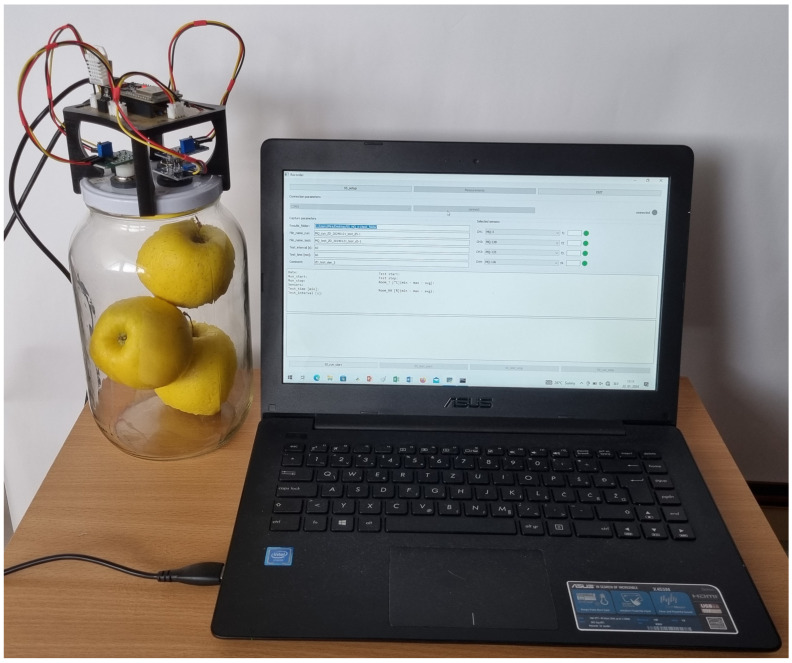
Experimental setup of electronic nose used in experiments.

Figure 2 shows the experimental set up of the electronic nose, which is positioned on the lid covering a glass jar (volume = 2.5 L) containing three apples. It is connected to the computer to collect and store sensor data.

The measurement process for each experiment performed on specific days was implemented with the following steps:

1. The electronic nose is connected to the power supply to allow sensors to stabilize and to the computer to run the initialization program. Run and test parameters are defined, including: (i) file names for storing measurements, (ii) selection of sensors (MQ3, MQ135, MQ136, and MQ138), and (iii) the measurement time (60 min). The start of the run phase is confirmed in the program.
2. Three apples are placed into the jar, which is then sealed with sensors on the cover. The start of the test phase is confirmed in the program.
3. The end of the test phase is confirmed. Data, defined as digital values calculated based on voltage response from the analog output of sensors, is stored in a .csv file.
4. The jar is opened, and the apples are removed to be used in the next analysis.
5. The end of the run phase is confirmed, and the electronic nose is prepared for the next test.

The data used in this study from the test phase consists of general parameters, including the date of the test and the minimum, maximum, and mean values of temperature and humidity measured in the environment. This is followed by five columns with consecutive numbers for sensor measurements (MQ3, MQ135, MQ136, and MQ138). The responses from the sensors are stored as digital values from voltage change, which is related to the concentration of VOCs. These values are calculated by the electronic nose control program as a twelve-bit value derived from the voltage levels sent by sensors (value = measurement [V] × 4096)/5 [V]).

### 3.3. Data Collection

Four experiments were performed using ‘Golden Delicious’ apples stored at ambient temperatures over two consecutive years, with one experiment performed in 2022 and three in 2023. The duration of each experiment varied due to limitations related to the availability of personnel and equipment in the faculty environment. Unfortunately, detailed information such as the harvest location, postharvest storage time, and specific storage conditions (temperature and humidity) during their time in the supermarket at the point of sale was not available. The objective was to collect sensor measurements from the electronic nose to define four datasets with five parameters (day, MQ3, MQ135, MQ136, and MQ138) for detailed analysis. The parameter day is included due to different sampling periods along several experiments used in further grouping of data. Four original datasets are as follows:

- GD(3–22)–12: Apples received direct from the cold store (T = 4 °C) from the Slovenian producers, weighing 554 g. Twelve (12) measurements were collected over 26 days (14 March 2022–8 April 2022) in intervals of two or three days (1, 3, 5, 8, 10, 12, 15, 17, 19, 22, 24, and 26). The apples were stored at an ambient temperature of T = 23 °C ± 2 °C and relative humidity RH = 31% ± 10%.
- GD(4–23)–8: Apples purchased at the supermarket (originating from Slovenia), weighing 589 g. Eight (8) measurements were collected over 23 days (3 April 2023–25 April 2023) in uneven intervals from one to four days (1, 2, 3, 8, 12, 17, 19, and 24). The apples were stored at an ambient temperature of T = 20 °C ± 2 °C and relative humidity RH = 45% ± 4%.
- GD(10–23)–16: Apples purchased at the supermarket (originating from Slovenia), weighing 570 g. Sixteen (16) measurements were collected over 36 days (4 October 2023–9 November 2023) in intervals of two, three, and four days (1, 3, 8, 10, 12, 14, 17, 19, 20, 22, 24, 26, 28, 32, 34, and 36). The apples were stored at an ambient temperature of T = 21 °C ± 2 °C and relative humidity RH = 62% ± 6%.
- GD(11–23)–16: Apples purchased at the supermarket (originating from Slovenia), weighing 591 g. Sixteen (16) measurements were collected over 36 days (7 November 2022–12 December 2022) in intervals of two, three, and four days (1, 3, 8, 10, 12, 14, 17, 19, 20, 22, 24, 26, 28, 32, 34, and 36). The apples were stored at an ambient temperature of T = 19 °C ± 1 °C and relative humidity RH = 55% ± 5%.

To standardize the datasets across all four experiments, measurements available on the same days (1, 3, 8, 12, 17, 19, and 24) were selected for further grouping into one larger dataset used in comparison. The new datasets were labeled as GD(3–22)–7, GD(4–23)–7, GD(10–23)–7, and GD(11–23)–7, each consisting of 7 measurements.

Due to the limited and varying number of days with available sensor measurements across experiments, six new datasets (Table 2) were defined by grouping original datasets based on specific characteristics related to time periods (autumn and spring), source of apples (cold store or supermarket), duration of experiments, and to the same day of the performed measurements. The objective was to create larger datasets for detailed analysis and explore generalized clustering and prediction models to determine ripening stages for training and evaluating new apple samples.

For the evaluation of ripening stages determined from classification analysis, completely new sensor measurements were collected using an electronic nose over nine days during four additional experiments (Test 1, Test 2, Test 3, and Test 4) performed in spring and autumn in years 2023 and 2024. These measurements were stored in a new dataset named TEST_S. All apples were purchased from a supermarket, and then they were stored and analyzed under conditions similar to those in the original experiments at an ambient temperature of T = 20 °C ± 3 °C and relative humidity RH = 45% ± 6%. To facilitate the review of classification performance, each sample of apples was labeled with the day of the performed measurements and the month of purchase, as shown in Table 3.

### 3.4. Methods and Analysis Approach

**Figure 3:**
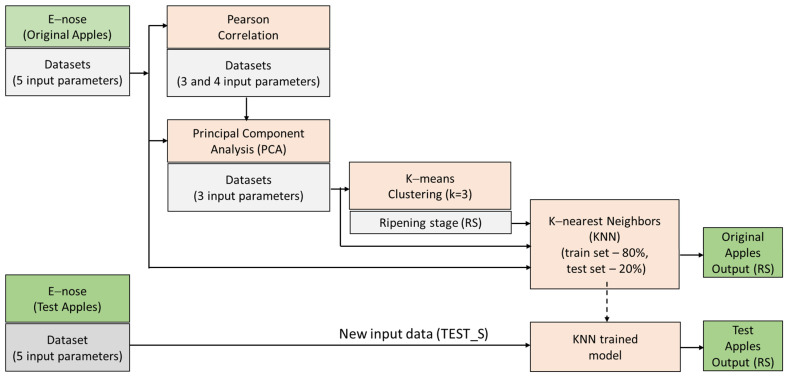
Diagram of an experimental approach with analysis methodology.

Figure 3 shows the diagram of the experimental setup, which encompasses three main steps: capturing sensor measurements, performing statistical and machine learning analysis, and evaluating the classification of ripening stages (RS). Pearson Correlation (PC) and Principal Component Analysis (PCA) were performed on ten datasets comprising 5 input parameters (day, MQ3, MQ135, MQ136, and MQ138) from specific days across four experiments using the electronic nose. Based on Pearson correlation, only 3 (day and two sensor parameters) and 4 input parameters (day and three sensor parameters) from the original datasets were used for the analysis of a smaller number of sensors in the electronic nose. The reduced new datasets defined by two principal components from PCA were constructed with 3 input parameters (day, PC1, and PC2) and employed in K-means clustering to determine ripening stages (RS). Datasets with 3 input parameters (day, PC1, and PC2) and original datasets with 5 input parameters (day, MQ3, MQ135, MQ136, and MQ138) along with previously defined ripening stages (RS) as output labels were utilized in the KNN classification method to perform the evaluation of ripening stages (RS) of original apple samples specified in the test set. A new dataset, TEST_S with 5 input parameters (day, MQ3, MQ135, MQ136, and MQ138), is used in the evaluation of ripening stages (RS) for new apple samples on a KNN-trained model.

Pearson correlation is a statistical measure with values between −1 and +1 used to analyze relationships between two variables, which is either positive (one variable increases, then the other also increases), negative (one variable increases, then the other decreases), or there is no correlation (one variable increases, then the other has no tendency to increase or decrease). It was employed to identify any linear relationships between various experiments or between sensors in the electronic nose.

Principal Component Analysis (PCA) is data preprocessing that reduces the number of dimensions by transforming potentially correlated variables into a smaller set of variables, called principal components [43]. These new variables are constructed as linear combinations of the original variables. PCA creates a dataset of uncorrelated variables to explore and summarize common characteristics. The first principal component explains the most variance in the data, while each subsequent component explains progressively less variance. The results from two principal components, PC1 and PC2, were used for K-means clustering to identify ripening stages. PCA analysis was performed using the Python programming tool.

K-means clustering is an unsupervised machine learning algorithm used in data analysis to group unlabeled instances [44]. Without prior training, unsorted data instances are organized into clusters based on shared characteristics, with each instance assigned to the cluster whose centroid is closest in terms of distance. The datasets resulting from PCA were analyzed with a specific number of clusters K = 3 to identify three ripening stages (less ripe, ripe, and overripe) for successive instances represented by the parameter day. K-means clustering was performed using Python 3.7.

The K-Nearest Neighbors (KNNs) is a supervised machine learning algorithm employed in classification problems [45]. It is particularly suitable for small datasets as a non-parametric method. KNN identifies the nearest neighbors in the training set based on a predefined parameter K, which is optimized using methods such as Cross-Validation or the Elbow method to determine the nearest neighbors closest in distance to the evaluated sample. It was performed using the KNN Python package.

To assess the effectiveness of the KNN model in determining the ripening stage of apples based on sensor measurements, the following well-known classification performance measures were used [46]:

- Accuracy (Acc) refers to closeness, as it provides the proportion of correctly classified samples to the total number of samples.
- Precision (Prec) focuses on the confidence measured by the ratio of correctly classified samples compared to the total number of samples predicted to belong to that class.
- Recall (Rec) presents a sensitivity defined as the ratio of correctly classified samples in proportion to the total number of samples in that class.
- The F1 score is a harmonic mean of precision and recall.

## 4. Results and Discussion

### 4.1. Sensor Measurements

#### 4.1.1. Data from Individual Experiments

**Figure 4:**
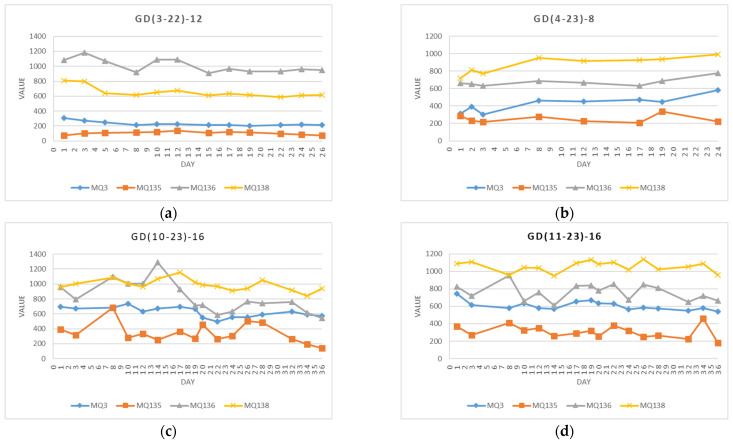
Sensor measurements in four experiments: (**a**) GD(3–22)–12: March-April 2022, 12 measurements for 26 days; (**b**) GD(4–23)–8: April 2023, 8 measurements for 23 days; (**c**) GD(10–23)–16: October and November 2023, 16 measurements for 36 days; and (**d**) GD(11–23)–16: November and December 2023, 16 measurements for 36 days.

The measurements from sensors MQ3, MQ135, MQ136, and MQ137 are presented separately for each experiment in Figure 4. Each line illustrates the signal value variations throughout the duration of the respective experiment. Notably, results for apples available in spring (GD(3–22)–12 and GD(4–23)–8), following a storage period of six to seven months, differ from those analyzed in autumn (GD(10–23)–16 and GD(11–23)–16), which were stored only for two months. It is observed that the changes in signal values over the investigated period are more stable for spring apples across all four sensors. In the GD(3–22)–12 experiment, signal values of sensor MQ136 are higher, and signal values of sensor MQ138 are lower compared to the other experiments. A plausible explanation for these results could be due to the origin and storage conditions of apples received directly from the producer, as well as differences in the experimental environment in 2022.

#### 4.1.2. Data from an Individual Sensor

**Figure 5:**
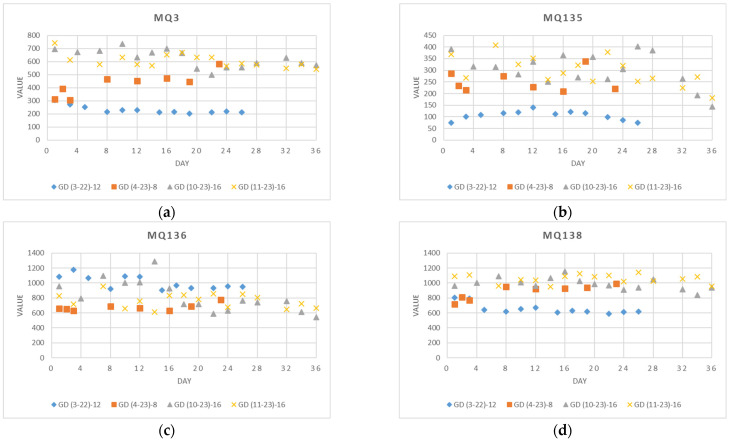
Sensor measurements: (**a**) MQ3, (**b**) MQ135, (**c**) MQ136, and (**d**) MQ138 received from experiments GD(3–22)–12, GD(4–23)–8, GD(10–23)–16, and GD(11–23)–16.

Figure 5 displays the varying numbers of measurements for sensors MQ3, MQ135, MQ136, and MQ138 in all four experiments, individually. In the experiment GD(3–22)–12, measurements from sensors MQ3, MQ135, and MQ138 exhibit notably lower mean, minimum, and maximum values than in experiments GD(10–23)–16 and GD(11–23)–16, whereas MQ136 shows slightly higher mean and minimum values. This indicates a clear differentiation of the origin and apple storage conditions before they were used in experiment GD(3–22)–12 in spring. Additionally, in the GD(4–23)–8 experiment performed in spring, signal values for all sensors MQ3, MQ135, MQ136, and MQ138 are slightly lower as compared to experiments GD(10–23)–16 and GD(11–23)–16, as they were all received from supermarkets but in a different period of the year. Very similar but higher values of sensor measurements are shown in experiments GD(10–23)–16 and GD(11–23)–16 in autumn, which is related to short storage time after the harvest before the performed analysis and corresponds to better sensory quality. The calculated standard deviation of measurements varied between 10% and 20%, which is quite high but can be explained by the various environmental conditions of temperature and humidity where apples were stored and experiments performed on different days.

**Figure 6:**
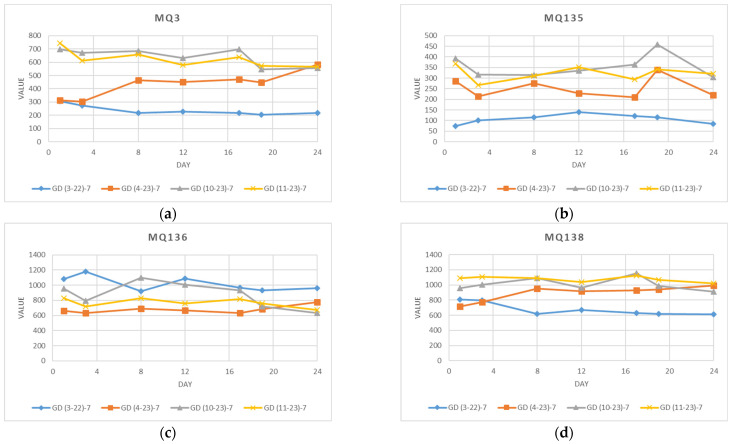
Sensor measurements ((**a**) MQ3, (**b**) MQ135, (**c**) MQ136, and (**d**) MQ138) from four experiments GD(3–22)–7, GD(4–23)–7, GD(10–23)–7, and GD(11–23)–7 on days 1, 3, 8, 12, 17, 19, and 24.

Considering all the collected data with different numbers of instances and performed on various days, along with the previously available information regarding the optimal quality of apples stored at ambient temperatures, it made sense to analyze only measurements on the same day over a duration of ays in all four experiments. For this purpose, only signal values from all four sensors available on days 1, 3, 8, 12, 17, 19, and 24 are shown in Figure 6.

#### 4.1.3. Test Data

**Figure 7:**
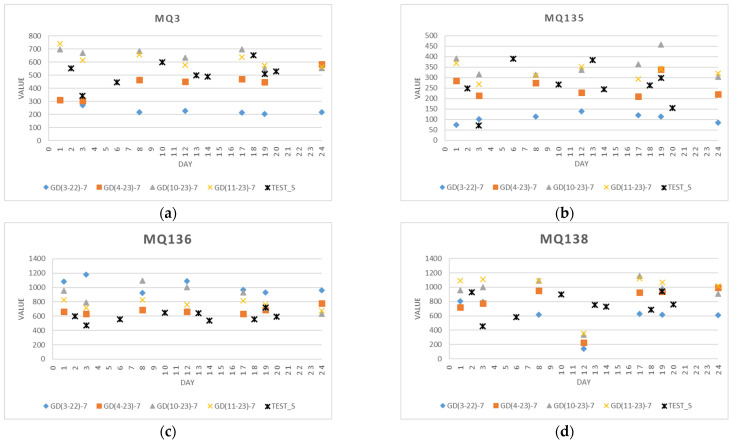
Sensor measurements ((**a**) MQ3, (**b**) MQ135, (**c**) MQ136, and (**d**) MQ138) from four test experiments defined as test sets (TEST_S) are added to the measurements from four experiments: GD(3–22)–7, GD(4–23)–7, GD(10–23)–7, and GD(11–23)–7 on days 1, 3, 8, 12, 17, 19, and 24.

A new dataset (TEST_S) was used for independent evaluation of ripening stages, regardless of the period and environmental conditions in which the basic experiments were carried out. Figure 7 shows the collected measurements from sensors MQ3, MQ135, MQ136, and MQ138 from the additional four experiments performed in 2023 and 2024. These measurements are visualized for comparison to the original datasets from experiments with seven instances: GD(3–22)–7, GD(4–23)–7, GD(10–23)–7, and GD(11–23)–7 on days 1, 3, 8, 12, 17, 19, and 24. The values of sensor measurements for the test samples of apples are quite similar to those of the initial experiments for the individual sensors.

### 4.2. Multivariate Analysis

#### 4.2.1. Pearson Correlation

Pearson correlation was performed to analyze the relationships between sensor parameters (MQ3, MQ135, MQ136, and MQ138) in individual experiments and the relationships between datasets (GD(3–22)–7, GD(4–23)–7, GD(10–23)–7, and GD(11–23)–7) for specific sensors used in electronic noses. A statistical test on datasets was conducted to determine the significance of the correlation.

Table 4 presents the comparison of correlation coefficients for sensors between datasets with various numbers of instances (GD(3–22)–12, GD(4–23)–8, GD(10–23)–16, and GD(11–23)–16) and datasets with a unified number of instances (GD(3–22)–7, GD(4–23)–7, GD(10–23)–7, and GD(11–23)–7). Additionally, calculated *p*-values for the first group of datasets are summarized in Table 5 to justify the significance of the presented result. For all datasets, sensors MQ3 and MQ138 show significant positive correlations with *p*-values lower than 0.05 (correlation coefficients are marked in bold). Sensors MQ3 and MQ136 have significant positive correlations with *p*-values lower than 0.05 in the experiments performed in the spring and in the experiment performed in October 2023. Sensors MQ136 and MQ138 have significant positive correlations with *p*-values lower than 0.05 in experiments performed in spring 2022 and in experiments performed in October 2023. Sensor MQ135 stands out with no significant correlation with other sensors for the four original datasets. The number of days on which measurements were collected does not substantially affect the correlation results of the sensors’ measurements.

Correlation analysis on the largest dataset GD(22–23)–all with 52 measurements and the dataset GD(22-23)–24d–7m with 28 measurements is included in Table 4. The significant correlations (*p*-values shown in Table 6) were identified for three pairs of sensors (MQ3 and MQ135), (MQ3 and MQ138), and (MQ135 and MQ138) with *p*-values lower than 1 × 10^−6^. For the pair of sensors (M135 and MQ136), there are significant correlations with a p-value lower than 0.5, and for the pair (MQ136 and MQ138), the *p*-value equals 0.045 for dataset GD(22–23)–all. There was no significant correlation between sensors MQ3 and MQ136, with a *p*-value of 0.071. Based on the presented correlation analysis of data from all experiments, either sensor MQ3 or MQ136 should be used in the electronic nose in combination with the other three sensors.

The presented results show significant correlation for all sensors except for MQ3 and MQ136 on datasets with measurements from all experiments, which is not the case in individual experiments. This could be explained by the release of VOCs during a limited period of one month on specific apple samples versus the period from October to March on various apple samples available for consumption.

Further examination of datasets GD(3–22)–7, GD(4–23)–7, GD(10–23)–7, and GD(11–23)–7 for individual sensors was performed to confirm any correlations, which would justify the use of newly specified datasets based on the season period, source of apple samples, and number of days for the analyses (Table 6).

For sensor MQ3, a considerably high correlation between experiments performed in the spring and autumn periods is identified. The significant correlation of −0.81 with a *p*-value of 0.026 is observed between two experiments GD(4–23)–7 and GD(3–22)–7 performed in spring when apples are more mature and for experiments GD(10–23)–7 and GD(11–23)–7 with a correlation coefficient of 0.76 with a *p*-value that amounts to 0.046 performed in autumn. This is attributed to the sensor’s sensitivity to alcohol, which is released as one of the major volatile organic compounds in apples during ripening.

For sensor MQ135, a considerably high correlation of 0.77 with a *p*-value amounting to 0.041 was found between two experiments, namely GD(10–23)–7 and GD(4–23)–7. For sensor MQ136, there is a considerably high correlation of 0.82 with a *p*-value of 0.024 that exists only between two experiments GD(10–23)–7 and GD(11–23)–7 for apples analyzed in autumn. For sensor MQ138, there is a considerably high correlation of −0.98 with a *p*-value of 1.4 × 10^−4^ for two experiments GD(3–22)–7 and GD(4–23)–7 and a lower correlation of 0.76 with a *p*-value of 0.046 for experiments GD(11–23)–7 and GD(10–23)–7.

Correlation analysis is crucial for understanding sensor relationships, thereby enhancing data interpretation, optimizing sensor systems for real-world applications, and exploring sensor fusion to combine data for effective representation of detected volatile organic compounds (VOCs) [31]. Electronic nose applications involve various commercial sensor systems and prototypes developed to evaluate different types of sensors measuring ethylene emissions [47] and study correlations with color parameters. These correlations were used to compare results across three ripening stages of pears (underripe, ripe, and overripe) determined using the firmness parameter.

#### 4.2.2. Principal Component Analysis

PCA was performed on each of the 10 datasets to derive reduced datasets using the principal components PC1 and PC2 for further determination of clusters. Raw signal values (MQ3, MQ135, MQ136, and MQ138) were preprocessed using a standard scaler in ‘Scikit-learn’, which normalizes data by removing the mean and scaling to unit variance. As part of the PCA analysis, factor loadings are presented in Table 7 for datasets GD(3–22)–12, GD(4–23)–8, GD(10–23)–16, and GD(11–23)–16 and Table 8 for datasets GD(22–23)–all, GD(22–23)–spring, GD(22–23)–autumn, and GD(22–23)–market. Factor loadings indicate the relative importance of individual sensors. The results for datasets GD(22–23)–24d and GD(22–23)–24d-7m are not presented as they only differed in the second decimal place as compared to GD(22–23)–all, from which both datasets originated. Notably, absolute values of factor loadings for sensors MQ3 and MQ138 remain consistent in all datasets, despite variations in instance sizes. Differences are observed for sensor MQ135 between datasets GD(22–23)–all and GD(22–23)–spring, while sensor MQ136 shows variation in the dataset GD(22–23)–all.

**Figure 8:**
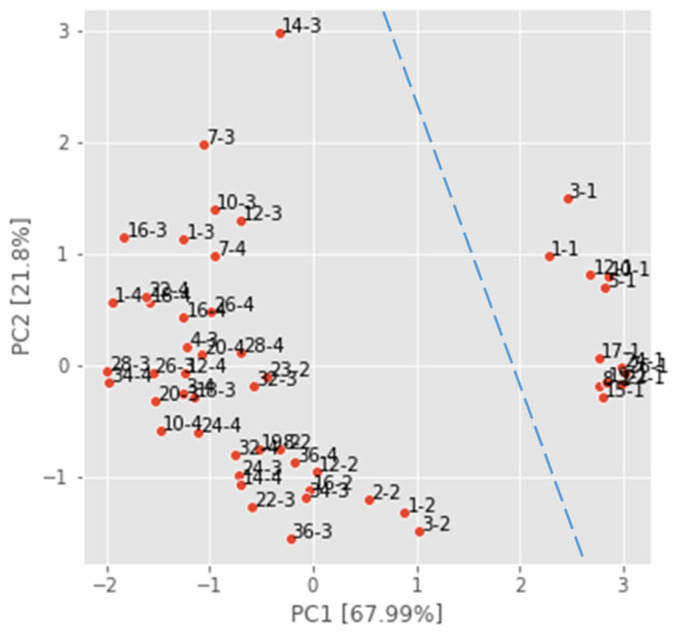
PCA analysis of dataset GD(22–23)–all (scaled values of instances are denoted with the day of performed measurement with added number: day–1 is for the dataset GD(3–22)–12; day–2 is for the dataset GD(4–23)–8; day–3 is for the dataset GD(10–23)–16; and day–4 is for the dataset GD(11–23)–16).

The scatter plot of the PCA analysis (Figure 8) illustrates the variance percentages captured by principal components PC1 (67.99%) and PC2 (21.8%) for the dataset GD(22–23)–all. The plot visualizes the clustering of sensor measurements across all instances. Notably, it distinguishes the apple samples received from the producer in the experiment GD(3–22)–12 on the right side of the graph (day–1) from those purchased at the supermarket in the other three experiments.

**Figure 9:**
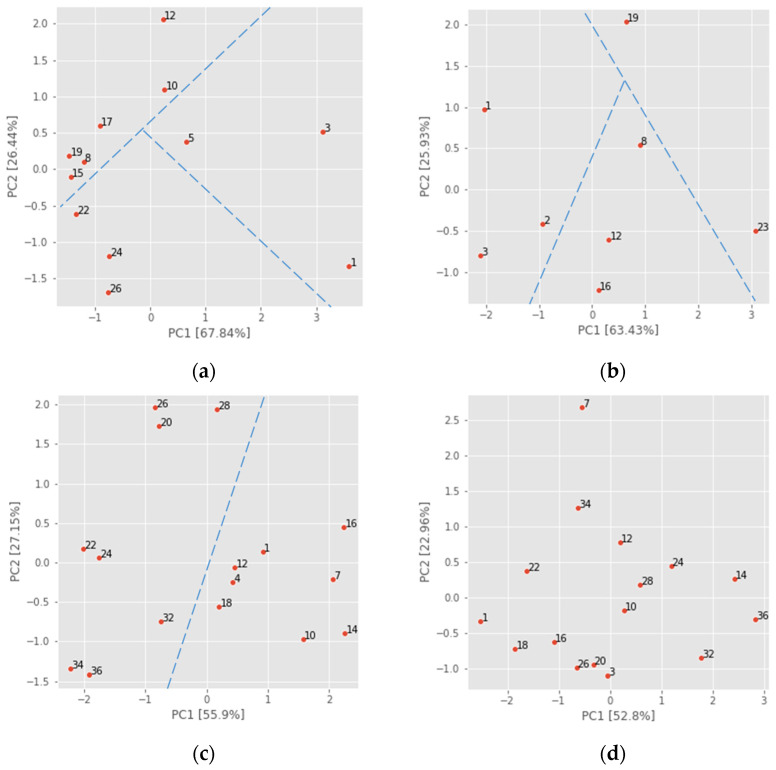
PCA analysis of four original datasets: (**a**) GD(3–22)–12, (**b**) GD(4–23)–8, (**c**) GD(10–23)–16, and (**d**) GD(11–23)–16.

Figure 9 shows percentage variations of principal components PC1 (ranging from 52.8% to 63.43%) and PC2 (ranging from 22.96% to 27.15%), alongside scaled values to visualize possible groups based on specific days of sensor measurements from individual experiments. The results show how the three possible groups for ‘Golden Delicious’ apples can be distinguished from datasets GD(3–22)–12 and GD(4–23)–8 (Figure 9a,b). These groups were estimated as degrees of ripening stages based on a study of computer vision systems for evaluating color parameters such as L*, a*, and b* in CIELab space [16], where PCA discriminated ripening stages according to days as unripe (1–8 day), ripe (9–31 day), and senescent (32–40 day) with 95.06% accuracy.

For the dataset GD(10–23)–16 (Figure 9c), it is possible to distinguish only two groups, while for the dataset GD(11–23)–16 (Figure 9d), it is not possible to clearly distinguish any grouping. Apples analyzed in autumn were generally less ripe, and signal data from sensors MQ3, MQ135, MQ136, and MQ138 were not sufficient to determine three ripening stages but only two ripening stages, as presented in [29], where fruits were classified only into two stages (fresh and corrupted). Additionally, an electronic nose with 12 MOS sensors [48] was used to differentiate between naturally ripe and artificially ripe crab apples based on signal values performed on PCA. The overlap was detected between both apple samples, which were not well clustered. In this study, apple samples from two distinct storage periods, after 1 month (autumn) and 6 months (spring), were selected in order to cover a broader period of time to measure VOCs. The PCA analysis shows the differences that were typically identified in other studies. The ripening stages were defined in advance, based on color or any other approach for successful classification of fruit ripening stages.

### 4.3. K-Means Clustering

K-means clustering was initially performed on sensor measurements from all four experiments presented in 10 previously described datasets that consist of five parameters (day, MQ3, MQ135, MQ136, and MQ138) to identify any common characteristics arising from differences in harvest location, storage time and conditions, handling within the supply chain, and the duration of the experiment. The parameter day was included due to different periods of measurement capture in the experiments. The clustering results were not satisfactory, as there was at least one instance, according to the time of the test (day), that did not fall into the expected cluster determined by consecutive days, which should be defined as unripe (0 − x days), ripe (x + 1 − y days), and overripe (y + 1 − 36). The variables x and y are expected to be defined with K-means clustering since the experiments were performed for up to 26 days in spring and up to 36 days in autumn.

Due to incomplete grouping with the original datasets, the principal components PC1 and PC2, obtained from the sensor measurements in PCA analysis, were used as new PCA datasets (denoted with extension: GD(3–22)–12)_PC, GD(4–23)–8_PC, …) with three parameters (day, PC1, and PC2). The K-means clustering method with parameter K = 3 (Table 9) provides a transparent set of clusters (G1(d), G2(d), and G3(d)) based on the available instances presenting specific consecutive days. These groups were then specified as three ripening stages (RS): less ripe (LR = G1(d)), ripe (R = G2(d)), and overripe (OR = G3(d)), including the missing days of measurements in the ripening stage to their end, as shown in the last three columns in Table 9.

Based on specified ripening stages such as LR, R, and OR with the same or very similar start day, it is possible to distinguish apples available to customers in spring (GD(3–22)–12_P and GD(4–23)–8_P) and in autumn (GD(10–23)–16_P and GD(11–23)–16_P). Furthermore, the results for apples analyzed in autumn (GD(22–23)–autumn_P) are similar to those for apples analyzed when purchased from the supermarket (GD(22–23)–market_P) and those collected from all four experiments (GD(22–23)–all_P). The ripening stages obtained for datasets with instances up to the 24th day (GD(22–23)–24d_P) and those with seven instances on the same day (GD(22–23)–24d–7m_P) differ from the others. Generally, there are some deviations from the start or end of each stage for one to two days.

Three ripening stages (LR, R, and OR) specified from the clustering results in Table 9 have been recognized and used for labeling the instances in datasets for further analysis. Comparable results of three ripening stages (unripe, ripe, and senescent) of ‘Golden Delicious’ apples were identified with PCA based on color CIE L*, a*, and b* parameters [16]. Another study presented an analysis of four ripening stages of banana fruit using data from a Dual MOS Electronic Nose to measure VOC parameters (aroma), achieving a classification accuracy of 98.10% with PCA and the KNN model [49].

### 4.4. KNN Classification

#### 4.4.1. Evaluation of KNN Training

The KNN classification model was developed to identify ripening stages (RS) of ‘Golden Delicious’ apple samples based on results from K-means clustering. The model used both the original datasets with five input parameters (day, MQ3, MQ135, MQ136, and MQ138) and the dataset with principal components PC1 and PC2, (day, PC1, and PC2). Raw sensor measurements were preprocessed using a standard scaler in ‘Scikit-learn’. For the training of KNN models, datasets were randomly split into two sets (train set −80% and test set −20%). The number of neighbors (K = 2, 3, 4) was selected using the Elbow method, considering the four different seasonal experiments and other datasets, with the largest containing 52 instances.

The accuracy measure showed good classification results, higher than 75% on both the train and test sets, even with 100% for the test set in datasets from experiment GD(3–22)–12 and both experiments in spring (GD(22-23)–spring). However, two datasets GD(4–23)–8 and GD(22–23)–24d–7m had worse accuracy results, likely due to the small number of instances in each ripening stage after splitting into training and test sets.

Classification results for the KNN model with principal components PC1 and PC2 showed an accuracy higher than 75% on both, the train and test sets in four datasets, with one dataset GD(3–22)–12_P achieving 100% accuracy for the test set. The other six datasets had lower accuracy results compared to the original datasets. The spring samples (GD(3-22)) generally showed higher accuracy, most probably due to more balanced VOCs in the late period after harvest compared to autumn samples.

#### 4.4.2. Evaluation of a New Dataset (TEST_S)

Table 10 presents the classification results of the KNN method on original datasets with five input parameters and a test dataset (TEST_S). For several datasets, more than one number of neighbors (K) yielded the same values of the measures Acc (%), Prec, Rec, and F1 score. The best accuracy equal to or higher than 78% (with only one or two misclassified instances) was achieved for three datasets (GD(22–23)–market, GD(22–23)–24d, and GD(22–23)–24d–7m) and equal to 100% for dataset GD(22–23)–all with measurements from all four experiments regardless of the number of instances.

Table 11 presents the classification results of the KNN method on datasets with three input parameters from PCA analysis (day, PC1, and PC2) and a test dataset (TEST_S). The best accuracy, equal to or higher than 78% (with only two misclassified instances), was achieved in eight datasets. The accuracy was lower only for datasets GD(4–23)–8_P and GD(11–23)–16_P, with three or four misclassified samples. For the dataset GD(22–23)–24d_P with measurements from all four experiments, regardless of the number of instances, the accuracy was 100%.

Table 12 shows the comparison of the misclassified ripening stages for instances in the test set (TEST_S) in the cases with the best results presented in Table 10 and Table 11. In two training sets (GD(22–23)–all and GD(22–23)–24d_P), all test instances were correctly classified. One instance, either from ripening stages ripe (R) and overripe (OR), was misclassified in six datasets (GD(22–23)–spring_P, GD(22–23)–market_P, GD(22–23)–24d, GD(22–23)–24d_P, GD(22–23)–24d–7m, and GD(22–23)–24d–7m_P). In five datasets, two or three instances were misclassified for training datasets (GD(3–22)–12_P, GD(4–23)–8, GD(10–23)–16_P, GD(11–23)–16_P, and (GD(22–23)–autumn_P). The less ripe (LR) stage was incorrectly classified as the ripening stage ripe (R), the ripe (R) stage was incorrectly classified as less ripe (LR), and the overripe (OR) stage was mistakenly classified as ripe (R).

When comparing the two types of datasets as seen in Table 12, it can be concluded that the highest accuracy of 100% (Err = 0%, meaning all apple samples are correctly classified) is achieved with the original dataset GD(22–23)–all and the dataset GD(22–23)–24d_P, which consists of parameters PC1 and PC1.

The KNN classification results of ripening stages presented in this study cannot be directly compared to other approaches analyzing surface features and color used with computer vision systems [14]. For detecting, grading, and classifying apples, a sensor array (MQ2, MQ3, MQ8, MQ6, MQ9, and MQ135) is used to collect data [50], which is then analyzed to predict or classify apple samples into ripening stages based on concentrations of VOCs. The PEN3 electronic nose was applied to detect VOCs in ‘Golden Delicious’ apples stored at 4 °C for 96 days [24]. They received R^2^ = 0.86 for the training set of the shelf life model and R^2^ = 0.98 to predict the shelf life. The results achieved by employing the PCA and KNN classification algorithms are in accordance with the use of an electronic nose to distinguish three ripening stages of banana: unripe, half-ripe, and full-ripe [49], with classification accuracy greater than 90%.

#### 4.4.3. Optimization of Number of Sensors

The correlation analyses provided an additional option for minimizing the number of sensors used in electronic nose experiments. It was established that sensors MQ3, MQ136, and MQ138 are satisfactory for determining ripening stages. Table 13 shows the accuracy results of training the original dataset on the test set (TEST_S) for a smaller number of sensors of two and three sensors compared to the results with four sensors. For each group of sensors, there exists at least one dataset for which all samples of apples are correctly classified into ripening stages. The best results with accuracy of 100% are achieved with results from the datasets with measurements from all experiments and the dataset from purchased apples.

Table 14 shows the comparison of accuracy results of training datasets with three input parameters (day, PC1, and PC2) on the evaluation on test set (TEST_S). The accuracy varies slightly, ranging from 78% to 100% for datasets with a larger number of instances when using two and three sensors.

When comparing the use of two types of datasets, it can be concluded that both provide satisfactory accuracies for each option of the selected number of sensors. There exist very similar results with better or equal classification accuracy results up to 100% for original datasets or datasets with principal components PC1 and PC2.

## 5. Conclusions

In this study, the ripening stages of ‘Golden Delicious’ apples during the unpredictable postharvest period and a fluctuating consumer environment with ambient temperatures were assessed using an electronic nose. This presented non-destructive method enables the measurement of volatile organic compounds (VOCs) during the ripening process of apples. Two experiments performed in spring (GD(3–22)–12 and GD(4–23)–8) represent apples after 7 months of storage, and two experiments in autumn (GD(10)–23)–16 and GD(11–23)–16 represent apples after two to three months of storage. All samples of apples were analyzed in years 2022 and 2023. For the experiment GD(3–22)–12, the apple samples were received directly from the producer, while for the other three experiments, the apples were purchased from a supermarket. At the beginning, sensor measurements of the electronic nose detecting volatile organic compounds were recorded for all experiments. Subsequently, correlation and PCA analysis were assessed to identify the minimal number of parameters and possible associations of data instances between experiments to define six new generalized datasets used for the determination of ripening stages. It was possible to identify the similarities of the experiments in terms of the post-harvest period related to the analysis in spring and in autumn and their source of origin when purchased at the supermarket or delivered from the producer. The duration of experiments for 24 or 36 days could also be an important optimization factor, with experiments performed on the same day but with different number of days between the execution of a single measurement with various sensors.

K-means clustering, as an unsupervised approach, based on data received from sensors MQ3, MQ135, MQ136, and MQ138, along with additional principal components PC1 and PC2 from PCA analysis, provided groups of instances for the determination of ripening stages. The original datasets with sensor measurements and datasets with principal components PC1 and PC2 were used for further classification of ripening stages with a supervised KNN model. Four additional experiments performed on specific days after the purchase of apples, defined as nine new test measurements, were successfully validated with accuracy higher than 75% and even 100% based on the results of 10 datasets from the original four experiments. The KNN model demonstrated successful separation of apples into distinct ripening stages. When evaluating the success of predicting a new sample of apples in new independent experiments, the highest number of misclassifications for less-ripe (LR), ripe (R), and overripe (OR) ripening stages for all train datasets from original experiments was detected, especially in autumn samples. By grouping data into larger datasets with similar time periods or even with different numbers of measurements used, the number of incorrect ripening stages is reduced to zero when all data are used. Additionally, regression and PCA analysis showed that measurements of volatile organic compounds obtained with only two or three sensors (MQ3, MQ136, and MQ138) also yielded adequate results from K-means clustering to successfully classify apples with the KNN model into three ripening stages, namely, LR, R, and OR, based on sensor measurements from four completely new experiments used for evaluation.

Based on these results, the consumers can significantly contribute to waste reduction, as they can be informed about the recommended optimal consumption time within the first eighteen days after purchase, when the apples are less ripe or ripe. After that period, the apples quality deteriorates. However, with some additional experiments in the future to collect a larger number of sensor measurements from uncovered periods after harvest and various places of origin, it would be possible to confirm the usefulness of the electronic nose for the determination of ripening stages. There is also an option to explore some other MOS sensors and further evaluate the time of measurement and provide the means to broaden their use across other fruits.

## Figures and Tables

**Table 1:** MOS sensors.

Name	Target Odors	Sensitivity Ranges (ppm)
MQ3	Alcohol (ethanol, methanol)	0.05–10
MQ135	NH_3_, benzene, hydrogen	10–10,000
MQ136	Hydrogen sulfide, sulfur	1–200
MQ138	Toluene, acetone, ethanol, hydrogen	5–500

**Table 2:** Six new datasets as defined by combining sensor measurements from four experiments (N—number of measurements).

New Dataset	Original Datasets	N
GD(22–23)–spring	GD(3–22)–12, GD(4–23)–8	20
GD(22–23)–autumn	GD(10–23)–16, GD(11–23)–16	32
GD(22–23)–market	GD(4–23)–8, GD(10–23)–16, GD(11–23)–16	40
GD(22–23)–all	GD(3–22)–12, GD(4–23)–8, GD(10–23)–, GD(11–23)–16	52
GD(22–23)–24d	GD(3–22)–12(11 *), GD(4–23)–8, GD(10–23)–16(11 *), GD(11–23)–16(11 *)	41
GD(22–23)–24d–7m	GD(3–22)–7, GD(4–23)–7, GD(10–23)–7, GD(11–23)–7	28

* Number of measurements selected from original datasets.

**Table 3:** Four experiments with nine new measurements stored in a dataset TEST_S.

Experiments	Purchase	Instance 1	Instance 2	Instance 3
Test 1	March 2023	(3–3)–3rd day		
Test 2	December 2023	(6–12)–6th day	(13–12)–13th day	(19–12)–19th day
Test 3	February 2024	(18–2)–18th day	(20–2)–20th day	
Test 4	March 2024	(2–3)–2nd day	(10–3)–10th day	(14–3)–14th day

**Table 4:** Pearson correlation matrix of the sensor parameters for comparison of two datasets (original dataset/dataset with fixed number of instances).

Experiment	Sensor	MQ3	MQ135	MQ136	MQ138
	MQ3	1			
GD(3–22)–12/	MQ135	−0.36/−0.61	1		
GD(3–22)–7	MQ136	**0.75/0.75**	0.07/−0.13	1	
	MQ138	**0.92/0.96**	−0.24/−0.46	**0.80/0.87**	1
	MQ3	1			
GD(4–23)–8/	MQ135	−0.12/−0.15	1		
GD(4–23)–7	MQ136	**0.75/0.74**	0.12/0.09	1	
	MQ138	**0.94/0.94**	−0.00/−0.05	0.60/0.58	1
	MQ3	1			
GD(10–23)–16/	MQ135	−0.08/−0.26	1		
GD(10–23)–7	MQ136	**0.71/0.75**	0.13/−0.25	1	
	MQ138	**0.51/0.59**	0.28/−0.00	**0.58/0.52**	1
	MQ3	1			
GD(11–23)–16/	MQ135	0.16/0.24	1		
GD(11–23)–7	MQ136	**0.42/0.74**	0.27/0.25	1	
	MQ138	**0.51/0.59**	0.28/−0.00	**0.58/0.52**	1
	MQ3	1			
GD(22–23)–all/	MQ135	**0.72/0.83**	1		
GD(22–23)–24d–7m	MQ136	−0.25/−0.22	**−0.29/−0.42**	1	
	MQ138	**0.92/0.94**	**0.72/0.77**	**−0.28**/−0.27	1

Bold indicates a significance value of *p* < 0.05.

**Table 5:** Calculated *p*-values for correlations of sensors.

Experiment	MQ3–MQ135	MQ3–MQ136	MQ3–MQ138	MQ135–MQ136	MQ135–MQ138	MQ136–MQ138
GD(3–22)–12	0.243	0.005	1.8 × 10^−5^	0.832	0.447	0.002
GD(4–23)–8	0.783	0.031	5 × 10^−4^	0.777	0.998	0.114
GD(10–23)–16	0.754	0.002	0.042	0.629	0.301	0.018
GD(11–23)–16	0.255	0.111	0.025	0.110	0.566	0.181
GD(22–23)–all	1.2 × 10^−9^	0.071	8.5 × 10^−23^	0.034	1.3 × 10^−9^	0.045
GD(22–23)–24d-7m	4.2 × 10^−8^	0.252	1.5 × 10^−13^	0.024	1.4 × 10^−6^	0.156

**Table 6:** Pearson correlation matrix of the datasets with 7 instances from the original datasets.

Sensor	Dataset	GD(3–22)–7	GD(4–23)–7	GD(10–23)–7	GD(11–23)–7
	GD(3–22)–7	1			
MQ3	GD(4–23)–7	**−0.81**	1		
	GD(10–23)–7	0.53	−0.57	1	
	GD(11–23)–7	0.71	−0.59	**0.76**	1
	GD(3–22)–7	1			
MQ135	GD(4–23)–7	−0.11	1		
	GD(10–23)–7	0.02	**0.77**	1	
	GD(11–23)–7	−0.11	0.54	0.48	1
	GD(3–22)–7	1			
MQ136	GD(4–23)–7	−0.51	1		
	GD(10–23)–7	0.05	−0.49	1	
	GD(11–23)–7	−0.19	−0.54	**0.82**	1
	GD(3–22)–7	1			
MQ138	GD(4–23)–7	**−0.98**	1		
	GD(10–23)–7	−0.25	0.14	1	
	GD(11–23)–7	0.38	−0.49	**0.76**	1

Bold indicates a significance value of *p* < 0.05.

**Table 7:** Factor loadings of PC1 and PC2 for sensors MQ3, MQ135, MQ136, and MQ138 on original datasets for four experiments.

Parameters	GD(3–22)–12		GD(4–23)–8		GD(10–23)–16		GD(11–23)–16	
	PC1	PC2	PC1	PC2	PC1	PC2	PC1	PC2
MQ3	**0.58**	−0.10	**0.61**	−0.12	**0.56**	−0.35	**−0.56**	−0.30
MQ135	−0.18	0.91	−0.01	0.97	0.15	0.91	−0.41	0.74
MQ136	**0.52**	0.39	**0.53**	0.19	**0.59**	−0.09	**−0.52**	0.29
MQ138	**0.58**	0.04	**0.58**	−0.03	**0.55**	0.21	**−0.50**	−0.56

Bold indicates similar PC1 values.

**Table 8:** Factor loadings of PC1 and PC2 for sensors MQ3, MQ135, MQ136, and MQ138 based on datasets by selecting measurements based on common characteristics.

Parameters	GD(22–23)–All		GD(22–23)–Spring		GD(22–23)–Autumn		GD(11–23)–Market	
	PC1	PC2	PC1	PC2	PC1	PC2	PC1	PC2
MQ3	**−0.57**	0.20	**0.52**	0.41	**0.58**	−0.32	**−0.57**	−0.27
MQ135	**−0.53**	0.08	**0.51**	−0.35	0.24	0.94	−0.33	0.93
MQ136	**0.27**	0.96	**−0.46**	0.65	**0.59**	−0.09	**−0.50**	−0.08
MQ138	**−0.57**	0.17	**0.50**	0.53	**0.49**	0.03	**−0.55**	−0.19

Bold indicates similar PC1 values.

**Table 9:** K-means results of clustering (K = 3) with parameters day, PC1, and PC2 for specific consecutive days used for the definition of ripening stages (LR = G1(d), R = G2(d), and OR = G3(d)).

PCA Dataset	G1(d)	G2(d)	G3(d)	LR = G1(d)	R = G2(d)	OR = G3(d)
GD(3–22)–12_P	1–5	8–18	19–26	1–7	8–18	19–26
GD(4–23)–8_P	1–3	8–12	16–23	1–7	8–15	16–23
GD(10–23)–16_P	1–12	14–24	26–36	1–13	14–25	26–36
GD(11–23)–16_P	1–12	14–24	26–36	1–13	14–25	26–36
GD(22–23)–spring_P	1–5	8–16	17–26	1–7	8–16	17–26
GD(22–23)–autumn_P	1–12	14–24	26–36	1–13	14–25	26–36
GD(22–23)–market_P	1–10	12–24	26–36	1–11	12–25	26–36
GD(22–23)–all_P	1–10	12–22	24–36	1–11	12–23	24–36
GD(22–23)–24d_P	1–8	10–17	18–26	1–9	10–17	18–26
GD(22–23)–24d–7m_P	1–3	8–17	19–24	1–7	8–18	19–24

**Table 10:** KNN classification results with Acc (%), Prec, Rec, and F1 scores for train datasets with 5 input parameters (day, MQ3, MQ135, MQ136, and MQ138) and test dataset (TEST_S) presented as (train/test), where the train result is defined by the smallest K with the best test results.

Dataset (5 Input Parameters)	K	Acc (%) (Train/Test)	Prec (Train/Test)	Rec (Train/Test)	F1 Score (Train/Test)
GD(3–22)–12	4	91.67/55.56	093/0.63	0.92/0.56	0.91/0.56
GD(4–23)–8	2	87.50/66.67	0.91/0.72	0.88/0.67	0.86/**0.66**
GD(10–23)–16	2	75.00/44.44	0.83/0.44	0.75/0.44	0.71/0.44
GD(11–23)–16	2	93.75/55.56	0.95/0.56	0.94/0.56	0.94/0.55
GD(22–23)–spring	4	85.00/55.56	0.86/0.69	0.85/0.56	0.85/0.56
GD(22–23)–autumn	2	87.50/66.67	0.90/0.70	0.88/0.67	0.87/0.67
GD(22–23)–market	2	85.00/77.78	0.88/0.78	0.85/0.78	0.84/0.78
GD(22–23)–all	4	82.59/100.00	0.84/1.00	0.83/1.00	0.82/**1.00**
GD(22–23)–24d	4	90.91/88.89	0.92/0.92	0.91/0.89	0.91/**0.89**
GD(22–23)–24d–7m	3	100.00/88.89	1.00/0.92	1.00/0.89	1.00/**0.89**

Bold indicates higher or equal values of the F1-score in comparison to the results in Table 11.

**Table 11:** KNN classification results for train datasets with 3 input parameters (day, PC1, and PC2) and test dataset—TEST_S) presented as (train/test), where the train result is the smallest K with the best results. The original name of the datasets is at the end marked with ‘_P’.

Dataset (3 Input Parameters)	K	Acc (%) (Train/Test)	Prec (Train/Test)	Rec (Train/Test)	F1 Score (Train/Test)
GD(3–22)–12_P	2	83.33/77.78	0.89/0.81	0.83/0.78	0.83/**0.77**
GD(4–23)–8_P	2	87.50/55.56	0.91/0.53	0.88/0.56	0.86/0.53
GD(10–23)–16_P	3	81.25/77.78	0.86/0.87	0.81/0.78	0.79/**0.78**
GD(11–23)–16_P	2	87.50/66.67	0.91/0.67	0.88/0.67	0.87/**0.66**
GD(22–23)–spring_P	2	90.00/88.89	0.92/0.92	0.90/0.89	0.90/**0.89**
GD(22–23)–autumn_P	2	87.50/77.78	0.90/0.78	0.88/0.78	0.87/**0.78**
GD(22–23)–market_P	3	92.50/88.89	0.93/0.92	0.92/0.89	0.93/**0.89**
GD(22–23)–all_P	3	90.38/100.00	0.91/1.00	0.90/1.00	0.90/**1.00**
GD(22–23)–24d_P	2	95.45/88.89	0.96/0.92	0.95/0.89	0.95/**0.89**
GD(22–23)–24d–7m_P	3	100.00/88.99	1.00/0.92	1.00/0.89	1.00/**0.89**

Bold indicates higher or equal values of the F1 score in comparison to the results in Table 10.

**Table 12:** KNN results of misclassified samples of apples (number of neighbors (K). For expected ripening stages, LR, R, and OR are shown as misclassified predicted ripening stages (day of test—month of purchase). N is the number of misclassified instances, and err (%) is the percentage of misclassified instances in the dataset TEST_S.

Dataset	K	LR	R	OR	N/(err %)
GD(3–22)–12_P	2	R(6–12)	OR(10–3)	0	2/(22.22)
GD(4–23)–8	2	R(2–3)	OR(13–12) OR(14–3)	0	3/(33.33)
GD(10–23)–16_P	3	R(3–3)	0	R(19–12)	2/(22.22)
GD(11–23)–16_P	2	0	OR(13–12) OR(14–3)	R(19–12)	3/(33.33)
GD(22–23)–spring_P	2	0	0	R(18–2)	1/(11.11)
GD(22–23)–autumn_P	2	R(3–3)	LR(13–12)	0	2/(22.11)
GD(22–23)–market_P	3	0	0	R(18–2)	1/(11.11)
GD(22–23)–all	4	0	0	0	0/(00.00)
GD(22–23)–all_P	3	0	0	0	0/(00.00)
GD(22–23)–24d	4	0	LR(13–12)	0	1/(11.11)
GD(22–23)–24d_P	2	0	OR(13–12)	0	1/(11.11)
GD(22–23)–24d–7m	3	0	OR(14–3)	0	1/(11.11)
GD(22–23)–24d–7m_P	3	R(3–3)	0	0	1/(11.11)

**Table 13:** KNN—Comparison of Acc (TEST_S) results for K neighbors with different combinations of measurements based on 2, 3, and 4 sensors MQ3, MQ136, and MQ138 on the original datasets. Bold numbers refer to higher accuracy in comparison with results in Table 11 with less than four apple samples misclassified.

Datasets	MQ (3–136) K/Acc (%)	MQ (3–138) K/Acc (%)	MQ (136–138) K/Acc (%)	MQ (3–136–138) K/Acc (%)	MQ (3–135–136–138) K/Acc (%)
GD(3–22)–12	**2/66.67**	**2/66.67**	**2/66.67**	**2/55.56**	4/55.56
GD(4–23)–8	**2/88.89**	**2/77.78**	3/88.89	2/77.78	**2/66.67**
GD(10–23)–16	3/66.67	2, 3, 4/55.56	3, 4/55.56	2/55.56	2/44.44
GD(11–23)–16	**4/77.78**	**4/88.89**	**2/100.00**	**4/88.89**	2/55.56
GD(22–23)–spring	**2, 4/88.89**	2, 4/77.78	4/77.78	2, 4/77.78	4/55.56
GD(22–23)–autumn	2, 4/66.67	2, 4/66.67	2/77.78	2, 3, 4/66.67	2/66.67
GD(22–23)–market	**2, 4/88.89**	**4/100.00**	**2/88.89**	**3, 4/88.99**	2/77.78
GD(22–23)–all	3/88.89	**4/100.00**	2, 3/77.78	**3, 4/100.00**	**4/100.00**
GD(22–23)–24d	**3/100.00**	2/77.78	**2, 3/88.89**	**3, 4/88.89**	**4/88.89**
GD(22–23)–24d–7m	**2, 3, 4/100.00**	**2, 3, 4/100.00**	2, 3/66.67	**3/100.00**	3/88.89

Bold indicates higher or equal values of the Acc measure in comparison to results in Table 14.

**Table 14:** KNN—comparison of Acc (TEST_S) results for K neighbors with different combinations of measurements based on 2, 3, and 4 sensors MQ3, MQ136, and MQ138 on the dataset with principal components PC1 and PC2.

Datasets	MQ (3–136) K/Acc (%)	MQ (3–138) K/Acc (%)	MQ (136–138) K/Acc (%)	MQ (3–136–138) K/Acc (%)	MQ (3–135–136–138) K/Acc (%)
GD(3–22)–12_P	2, 3, 4/44.44	2, 3, 4/44.44	2/55.56	2/55.56	**2/77.78**
GD(4–23)–8_P	**2/88.89**	3, 4/55.56	**3/100.00**	2, 3, 4/88.89	2/55.56
GD(10–23)–16_P	**2, 3, 4/77.78**	**3/77.78**	**2, 3/66.67**	**2, 4/77.78**	**3/77.78**
GD(11–23)–16_P	**2, 3, 4/77.78**	4/77.78	3, 4/88.89	3/77.78	**2/66.67**
GD(22–23)–spring_P	2/77.78	**2, 3/88.89**	**2, 4/77.78**	**2/88.89**	**2/88.89**
GD(22–23)–autumn_P	**2, 3, 4/66.67**	**3, 4/66.67**	**2, 4/88.89**	**2, 4/77.78**	**2/77.78**
GD(22–23)–market_P	2, 3, 4/77.78	**2/100.00**	**2/88.89**	**2, 4/88.89**	**3/88.89**
GD(22–23)–all_P	3, 4/**100.00**	3/88.89	**2, 3, 4/88.89**	**3, 4/100.00**	**3/100.00**
GD(22–23)–24d_P	**3/100.00**	**3/100.00**	**2, 3, 4/88.89**	**4/88.89**	**2/88.89**
GD(22–23)–24d–7m_P	2, 3, 4/77.78	2, 3, 4/77.78	**3/88.89**	2, 3/77.78	3/**88.99**

Bold indicates higher or equal values of the Acc measure in comparison to results in Table 13.

## Data Availability

The original contributions presented in the study are included in the article, further inquiries can be directed to the corresponding author.
